# Comparative Diagnostic Performance of Metabolic Scoring Indices for Ultrasonographic Hepatic Steatosis: Development and Validation of a Novel Composite Score in 1204 Consecutive Patients

**DOI:** 10.3390/jcm15114366

**Published:** 2026-06-04

**Authors:** Gülşah Altun, Enver Çiftel

**Affiliations:** 1 Department of Internal Medicine, Sivas Numune Hospital, 58100 Sivas, Turkey; 2 Department of Endocrinology and Metabolism, Sivas Numune Hospital, 58100 Sivas, Turkey

**Keywords:** metabolic dysfunction-associated steatotic liver disease, MASLD, hepatic steatosis, TyG index, lipid accumulation product, visceral adiposity index, composite score, ultrasonography, AUROC, logistic regression, biomarkers

## Abstract

**Background/Objectives**: Metabolic dysfunction-associated steatotic liver disease (MASLD), formerly termed non-alcoholic fatty liver disease (NAFLD), is the most prevalent chronic liver condition globally, affecting one-quarter of the adult population. Non-invasive metabolic indices offer a pragmatic alternative to liver biopsy for population-level steatosis screening. We aimed to systematically compare the diagnostic performance of four widely used metabolic indices—TyG, LAP, FLI, and VAI—and to derive a novel composite score that demonstrably surpasses each constituent index. **Methods**: Retrospective cross-sectional study; 1204 consecutive adults undergoing abdominal ultrasonography at a single tertiary centre (study period: 2024–2025; data collection period: January–April 2026); steatosis graded 0–3 by certified radiologists; four metabolic indices retrieved from pre-calculated columns; novel composite score derived by binary logistic regression with 10-fold stratified cross-validation (stratified by binary outcome); AUROC with 1000-iteration bootstrap 95% CI; and pairwise comparisons by the DeLong method. **Results**: Of 1204 patients (mean age 42.3 ± 12.5 years; 73.3% male; 13.4% with diabetes), steatosis grades were: Grade 0, 185 (15.4%); Grade 1, 539 (44.8%); Grade 2, 311 (25.8%); and Grade 3, 169 (14.0%). For significant steatosis (Grade ≥ 2), TyG achieved AUROC 0.884 [0.863–0.904] and LAP 0.883 [0.862–0.901], while FLI showed only moderate performance. The novel composite score achieved AUROC 0.896 [0.870–0.920] for any steatosis and 0.922 [0.906–0.938] for significant steatosis, significantly outperforming all four individual indices (DeLong *p* = 0.004 vs. TyG; *p* = 0.002 vs. LAP; *p* < 0.001 vs. FLI; *p* < 0.001 vs. VAI). Youden-optimal performance: sensitivity 87.1%, specificity 86.0%, and Youden index 0.731. **Conclusions**: A 10-fold cross-validated logistic composite of four metabolic indices significantly outperforms each constituent index for ultrasonographic hepatic steatosis detection. The composite score may serve as a practical, non-invasive tool for steatosis risk stratification pending external validation.

## 1. Introduction

Metabolic dysfunction-associated steatotic liver disease (MASLD), the nomenclature adopted by international consensus in 2023 to replace non-alcoholic fatty liver disease (NAFLD), is the most common chronic liver disorder globally, with a pooled prevalence of approximately 25% in the general adult population and steeply rising incidence driven by the dual epidemics of obesity and type 2 diabetes mellitus [1,2]. The MASLD spectrum encompasses a continuum from simple hepatic steatosis through metabolic dysfunction-associated steatohepatitis (MASH), progressive fibrosis, and cirrhosis, with substantial attributable risk for hepatocellular carcinoma, end-stage liver disease, and premature cardiovascular mortality [3,4,5]. Throughout this manuscript, the term NAFLD is retained where it refers specifically to the original derivation populations of the four scoring indices under study, each of which was developed and validated prior to the 2023 nomenclature revision; all contemporary references employ MASLD terminology.

Liver biopsy, the reference standard for hepatic steatosis grading and fibrosis staging, is precluded from routine population-level screening by its invasive nature, procedural risk, and sampling variability [4]. Abdominal ultrasonography (USG) has therefore become the de facto first-line imaging modality: it is widely accessible, free of ionising radiation, and achieves a diagnostic sensitivity range of 65–89% and specificity range of 80–93% for moderate-to-severe steatosis [6,7]. However, its performance degrades substantially for mild steatosis (grade 1), where hepatic fat deposition < 30% may fall below the ultrasonographic detection threshold, and its operator-dependent and semi-quantitative nature limits reproducibility [7,8].

These limitations have motivated the development of non-invasive metabolic scoring indices that exploit routinely available biochemical and anthropometric parameters. Four indices have accumulated the most evidence:

**TyG index (Triglyceride–Glucose Index):** a surrogate of hepatic insulin resistance computed as the natural logarithm of fasting triglycerides × fasting glucose/2 [8];

**LAP score (Lipid Accumulation Product):** a sex-specific product of waist circumference and fasting triglycerides that quantifies visceral lipid excess [9];

**FLI (Fatty Liver Index):** a four-component index incorporating BMI, waist circumference, triglycerides, and GGT, originally developed in the Dionysos cohort [10];

**VAI (Visceral Adiposity Index):** a sex-specific formula integrating waist circumference, BMI, triglycerides, and HDL cholesterol to estimate visceral adipose function [11].

Despite the rapidly growing literature, three fundamental problems remain unresolved. First, comparative performance across populations is inconsistent: the index achieving the highest AUROC in one cohort often underperforms in another, with ethnic composition, BMI distribution, and reference standard choice explaining substantial variance [11,12,13,14]. Second, no published study has simultaneously compared all four indices in a single large cohort, precluding direct conclusions about relative superiority. Third, and most importantly, the assumption that no linear combination of these indices can outperform the best individual score has not been formally tested with appropriate cross-validation methodology.

We address all three gaps with a consecutively enrolled cohort of 1204 patients—the largest single-centre comparison of these four indices to date—and present the following contributions: (1) a comprehensive head-to-head comparison of TyG, LAP, FLI, and VAI against ultrasonographic steatosis grading; (2) identification of the best-performing individual index; and (3) derivation and internal validation of a novel composite score demonstrably superior to each constituent index.

## 2. Patients and Methods

### 2.1. Study Design and Ethical Approval

This retrospective, single-centre, cross-sectional study was conducted at the Internal Medicine outpatient clinics of Sivas Numune Hospital, Sivas, Turkey. The study protocol was reviewed and approved by the Sivas Cumhuriyet University Health Sciences Research Ethics Committee (approval number: 25_12_11_41; meeting date: 11 December 2025), prior to the commencement of retrospective data collection. Patient records from the study period (2024–2025) were retrieved and analysed between January and April 2026. All procedures were conducted in conformity with the ethical principles of the Declaration of Helsinki (revised 2013). Because this study retrospectively analysed deidentified clinical records, informed consent was waived by the ethics committee. This study is reported in accordance with the STARD 2015 (Standards for Reporting of Diagnostic Accuracy Studies) guidelines [15].

### 2.2. Participants

Adults aged ≥18 years who had attended internal medicine, endocrinology, family medicine, or obesity clinics and had undergone abdominal ultrasonography during the study period (2024–2025), and whose records were retrospectively retrieved during the data collection period (January–April 2026), were considered for eligibility. The following patients were excluded: (i) known chronic liver disease including viral hepatitis (HBV, HCV), autoimmune hepatitis, Wilson disease, hereditary haemochromatosis, or alpha-1 antitrypsin deficiency; (ii) established cirrhosis, portal hypertension, or acute hepatic failure; (iii) alcohol consumption exceeding 20 g/day (women) or 30 g/day (men), or history of alcohol-use disorder; (iv) current use of hepatotoxic agents (e.g., amiodarone, tamoxifen, long-term systemic corticosteroids); (v) pregnancy or lactation; (vi) eGFR < 30 mL/min/1.73 m^2^; and (vii) unavailability of any metabolic parameter required for index calculation within the same clinical encounter. After applying all exclusion criteria, a final cohort of *n* = 1204 patients was enrolled.

### 2.3. Ultrasonographic Assessment

All B-mode abdominal ultrasonographic examinations were performed in the Radiology Department of Sivas Numune Hospital using a Philips EPIQ Elite ultrasound system (Philips Healthcare, Bothell, WA, USA) by board-certified radiologists blinded to metabolic parameters. Hepatic steatosis was classified on an established four-point ordinal scale: Grade 0 (absent: normal parenchymal echogenicity with clearly visualised intrahepatic vasculature and diaphragm); Grade 1 (mild: mildly increased parenchymal echogenicity with preserved diaphragm and portal vein wall visualisation); Grade 2 (moderate: markedly increased echogenicity with partially impaired diaphragm and portal vein wall definition); and Grade 3 (severe: markedly increased echogenicity with poor or absent diaphragm visualisation and loss of intrahepatic vascular definition). This grading system corresponds to the widely cited Hamaguchi criteria [7]. Because this study analysed retrospectively archived USG reports from multiple radiologists, a prospective inter-reader reliability assessment was not performed; this constitutes a pre-specified limitation that is addressed in Section 4.

### 2.4. Metabolic Scoring Indices

All four metabolic indices were extracted from the clinical database, where they had been pre-computed at the time of patient registration; reference formulae are provided below for transparency and reproducibility [8,9,11,16]:

**TyG index** = ln[fasting triglycerides (mg/dL) × fasting glucose (mg/dL)/2].

**LAP score** = Males: [WC (cm) − 65] × TG (mmol/L); Females: [WC − 58] × TG (mmol/L).

**FLI** = {exp[0.953 × ln(TG) + 0.139 × BMI + 0.718 × ln(GGT) + 0.053 × WC − 15.745]}/{1 + exp[…]} × 100.

**VAI** = Males: [WC/(39.68 + 1.88 × BMI)] × [TG/1.03] × [1.31/HDL]; Females: [WC/(36.58 + 1.89 × BMI)] × [TG/0.81] × [1.52/HDL] (mmol/L).

### 2.5. Novel Composite Score: Derivation and Validation

The novel composite score was derived using binary logistic regression, with all four metabolic indices as continuous predictors. Two distinct models were fitted corresponding to two clinically meaningful diagnostic endpoints:

**Model 1**—Detection of any hepatic steatosis: Grade 0 (reference) vs. Grades 1–3.

**Model 2**—Detection of significant hepatic steatosis: Grades 0–1 (reference) vs. Grades 2–3. This endpoint is of greater clinical relevance as Grade ≥ 2 steatosis is associated with substantially elevated cardiometabolic risk, higher likelihood of concomitant NASH, and stronger indications for pharmacological and lifestyle intervention.

All predictors were standardised to zero mean and unit variance (z-score normalisation) prior to regression. This ensures that resulting regression coefficients are directly interpretable as the effect of a one-standard-deviation increase in each index, facilitating the comparison of relative predictor importance.

To generate unbiased, out-of-sample performance estimates and guard against overfitting—a critical methodological requirement when developing a composite score in the same dataset used for evaluation—10-fold stratified cross-validation (stratified by the binary significant-steatosis outcome [Grade ≥ 2], preserving outcome prevalence across folds) was applied throughout model development using a Pipeline that fits the StandardScaler exclusively on training folds, ensuring no information from test folds influences standardisation. In each cross-validation cycle, the model was fitted on 90% of the data and predicted probabilities were generated for the remaining 10%. The final composite score value for each patient therefore represents a held-out prediction from a model that never observed that patient during training. All reported AUROC values and diagnostic performance metrics were computed exclusively from these cross-validated, out-of-sample predictions.

For reference, the composite score formula corresponding to the significant steatosis model (Model 2) can be expressed as a linear combination of standardised predictors:

log-odds = 0.000 + (+0.9444 × z_TYG) + (−0.0749 × z_LAP) + (+1.1156 × z_FLI) + (+2.9025 × z_VAI)

Composite probability = 1/(1 + exp(−log-odds))

where z_X denotes the standardised (z-score transformed) value of score X using the population mean and standard deviation of the derivation sample. For clinical applicability, population means and standard deviations (estimated on the full derivation cohort) are provided, enabling the prospective application of the composite score formula to individual patients.

### 2.6. Statistical Analysis

Continuous variables were tested for normality using the Shapiro–Wilk test. All metabolic indices showed non-normal distributions (all Shapiro–Wilk *p* < 0.001), consistent with right-skewed distributions expected for metabolic risk biomarkers. Non-normally distributed continuous variables are presented as median (interquartile range; IQR) and compared across four USG grade groups using the Kruskal–Wallis test. Post hoc multiple comparisons were performed using the Dunn test with Bonferroni correction for all six grade-pair combinations.

Diagnostic accuracy was assessed by constructing ROC curves for each metabolic index and the composite score against two binary endpoints (any steatosis; significant steatosis). The AUROC with 95% CI was the primary performance metric. Bootstrap confidence intervals were computed using 1000 resampling iterations. Pairwise AUROC comparisons between the composite score and each individual index were performed using the DeLong method [17]. Individual indices (TyG, LAP, FLI, VAI) are defined by fixed, parameter-free formulae; their AUROCs are identical whether evaluated on the full dataset or out-of-fold subsets (empirical difference < 0.002 across all four indices), rendering DeLong comparisons against the cross-validated composite score methodologically valid. The Youden index (J = sensitivity + specificity − 1) was used to identify the optimal diagnostic cut-off.

Model calibration was assessed graphically using calibration curves (observed vs. predicted probability, 10 quantile-based bins) and quantified using the Hosmer–Lemeshow chi-square statistic. Composite score inter-component correlations were assessed by Spearman rank correlation, and the correlation of each component and the composite score with USG grade was characterised by Spearman r. Categorical variables are expressed as frequency (%) and compared by chi-square test. All analyses were performed using Python 3.11 (NumPy 1.26, SciPy 1.11, scikit-learn 1.4, pandas 2.1). The composite score model employed a scikit-learn Pipeline to ensure that StandardScaler parameters were estimated exclusively from training-fold data in each iteration, precluding data leakage from test observations. The composite score model used a scikit-learn Pipeline object to ensure StandardScaler parameters were estimated exclusively from training-fold data in each cross-validation iteration, precluding any form of data leakage from test folds. Statistical significance was set at *p* < 0.05 (two-tailed); no correction for multiple comparisons was applied across the primary ROC analyses, but Bonferroni correction was applied to post hoc grade-pair comparisons.

Multicollinearity among the four predictors was quantified using the Variance Inflation Factor (VIF), calculated from the correlation matrix of standardised predictors. VIF values are reported as a model diagnostic.

Decision curve analysis (DCA) was performed to evaluate the clinical net benefit of the composite score and the best-performing individual indices (TyG, LAP) across a range of threshold probabilities (0.05–0.70) for the detection of significant steatosis, using the cross-validated composite score predictions.

## 3. Results

### 3.1. Patient Characteristics

The final cohort comprised 1204 patients with a mean age of 42.3 ± 12.5 years; 73.3% were male and 13.4% had diabetes mellitus. The distribution of USG hepatic steatosis grades was: Grade 0 (no steatosis) in 185 patients (15.4%), Grade 1 (mild) in 539 (44.8%), Grade 2 (moderate) in 311 (25.8%), and Grade 3 (severe) in 169 (14.0%). Thus, 84.6% of the cohort had at least some degree of steatosis and 39.8% had significant steatosis (Grade ≥ 2). Baseline clinical and biochemical characteristics stratified by USG grade are presented in Table 1.

All continuous variables differed significantly across USG grades (Kruskal–Wallis *p* < 0.001 for all). Progressive increases were observed in fasting glucose (89.4 to 135.8 mg/dL), triglycerides (83.5 to 273.3 mg/dL), ALT (22.0 to 30.7 U/L), GGT (17.8 to 35.4 U/L), and waist circumference (99.8 to 116.6 cm) from Grade 0 to Grade 3, with a reciprocal progressive decline in HDL cholesterol (53.5 to 42.6 mg/dL). Prevalence of male sex (61.6% at Grade 0 vs. 81.1% at Grade 3, *p* < 0.001) and diabetes mellitus (1.1% vs. 36.1%, *p* < 0.001) increased substantially across grades, underscoring the metabolic burden associated with higher steatosis grades.

### 3.2. Metabolic Index Values by Steatosis Grade

**Figure 1:**
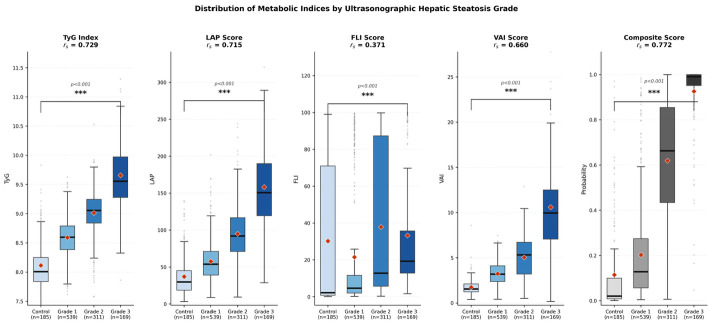
Distribution of metabolic indices and the novel composite score by ultrasonographic hepatic steatosis grade. Boxes represent IQR; horizontal line = median; diamond = mean; whiskers = 5th–95th percentiles. Spearman rank correlation coefficient (r) with USG grade is shown for each panel. All four indices and the composite score show significant grade-associated increases (all Kruskal–Wallis *p* < 0.001). *** *p* < 0.001 Grade 0 vs. Grade 3 (Bonferroni-corrected Dunn test). Values are median (interquartile range). r denotes Spearman rank correlation coefficient with USG grade (all *p* < 0.001). Post hoc pairwise comparisons by Dunn test with Bonferroni correction: all grade pairs significant (*p* < 0.001) for TyG, LAP, and VAI. FLI, Fatty Liver Index; LAP, Lipid Accumulation Product; TyG, Triglyceride–Glucose index; VAI, Visceral Adiposity Index.

Median values of TyG, LAP, and VAI increased monotonically from Grade 0 to Grade 3, confirming their grade-dependent biological signal (all Kruskal–Wallis *p* < 0.001; Table 2; Figure 1). TyG showed a near-consistent increment per grade step (0.59, 0.46, and 0.50 units respectively) per grade step (Grade 0: 8.01, Grade 1: 8.60, Grade 2: 9.05, Grade 3: 9.55). LAP demonstrated the most dramatic absolute gradient, spanning 5.1-fold from Grade 0 (median 29.72 [IQR 18.63–45.12]) to Grade 3 (150.66 [119.50–190.04]), reflecting the compounded effect of progressive central adiposity and dyslipidaemia. VAI increased 6.4-fold from Grade 0 (1.55 [1.23–2.10]) to Grade 3 (9.95 [7.07–12.50]).

Spearman rank correlations with USG grade were strongest for TyG (r = 0.729, *p* < 0.001), LAP (r = 0.715, *p* < 0.001), and VAI (r = 0.660, *p* < 0.001). FLI showed a substantially weaker association (r = 0.371, *p* < 0.001). Post hoc Dunn comparisons (Bonferroni-corrected) confirmed significant differences between every possible pair of grades for TyG, LAP, and VAI (all *p* < 0.001). The novel composite score achieved the strongest grade-correlation of all metrics (r = 0.772, *p* < 0.001).

### 3.3. Diagnostic Performance of Individual Metabolic Indices

**Figure 2:**
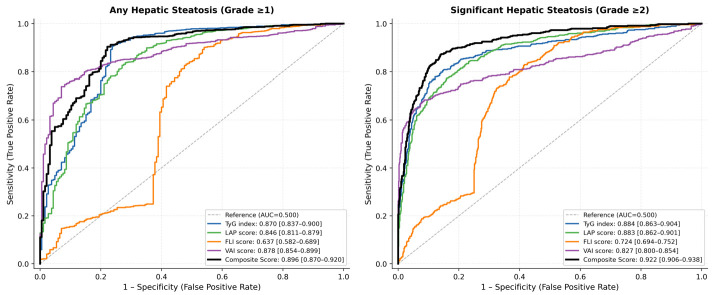
ROC curves for the detection of significant hepatic steatosis (Grade ≥ 2 vs. Grade 0–1). Left panel: full ROC space. Right panel: zoomed view of the high-sensitivity region. The novel composite score (solid black line) demonstrates consistently higher true positive rates across all false positive rate thresholds compared with each individual index.

For the detection of any hepatic steatosis (Grade 0 vs. Grades 1–3), AUROCs were: VAI 0.878 [0.854–0.899], TyG 0.870 [0.837–0.900], LAP 0.846 [0.811–0.879], and FLI 0.637 [0.582–0.689]. The three leading indices (VAI, TyG, LAP) demonstrated excellent discrimination, while FLI showed only moderate performance. For the clinically more important endpoint of significant steatosis (Grade ≥ 2 vs. Grade 0–1), TyG led with AUROC 0.884 [0.863–0.904], closely followed by LAP 0.883 [0.862–0.901], with VAI slightly lower at 0.827 [0.800–0.854]. FLI (0.724 [0.694–0.752]) showed a moderate performance. Full ROC curves are shown in Figure 2.

### 3.4. Novel Composite Score: Performance and Validation

**Figure 3:**
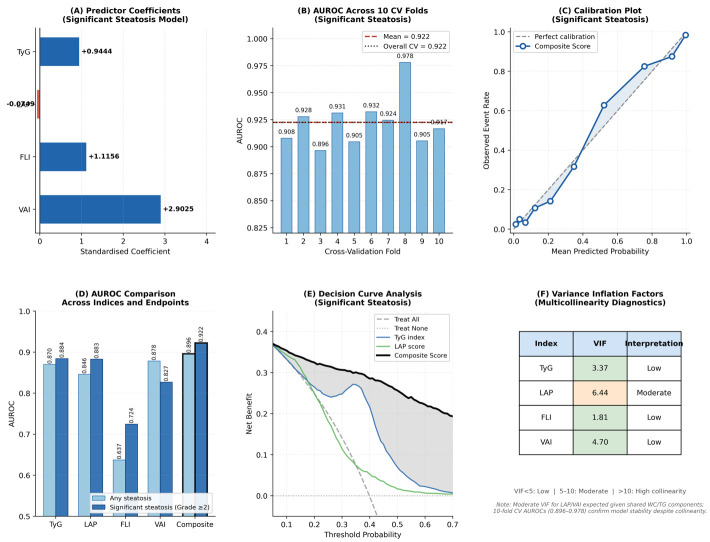
Composite score development and internal validation. (**A**) Standardised logistic regression coefficients for the significant steatosis model; bar length reflects relative predictor weight. (**B**) AUROC across 10 cross-validation folds demonstrating model stability (range 0.896–0.978). (**C**) Calibration plot: observed vs. predicted probability; proximity to the diagonal indicates good calibration. (**D**) AUROC comparison across all scores and both endpoints. (**E**) Decision curve analysis: net benefit of the composite score and best-performing individual indices across clinically relevant threshold probabilities. (**F**) Variance Inflation Factors confirming low-to-moderate multicollinearity among the four predictors. In panel (**F**), green indicates low (VIF < 5) and orange indicates moderate (VIF 5–10) collinearity.

Standardised logistic regression coefficients for the significant steatosis model (Model 2) are presented in Figure 3A. The largest contribution was from VAI (β = +2.9025), followed by FLI (β = +1.1156) and TyG (β = +0.9444), with LAP (β = −0.0749) playing a minor role. The negative LAP coefficient reflects multicollinearity with VAI and TyG; once both are included, the marginal contribution of LAP is absorbed. This is expected behaviour in correlated predictor systems and does not imply inverse association when LAP is examined alone. Importantly, LAP remains strongly and positively associated with steatosis grade when evaluated individually (Spearman r = 0.715, *p* < 0.001; Table 2); the negative sign is a multivariate modelling artefact, not a reversal of the bivariate relationship.

Cross-validated AUROC for the composite score was 0.896 [0.870–0.920] for any steatosis and 0.922 [0.906–0.938] for significant steatosis. The 10-fold cross-validation fold AUROCs for significant steatosis ranged from 0.896 to 0.978, demonstrating consistent model stability across all folds (Figure 3B). At the Youden-optimal cut-off of 0.379 for significant steatosis, the composite score achieved sensitivity 87.1%, specificity 86.0%, and Youden index 0.731—superior to the best individual index at its own optimal cut-off. The calibration plot (Figure 3C) confirmed a good agreement between predicted probabilities and observed event rates across the full probability range, indicating that the composite score is well-calibrated and its output is interpretable as genuine probability estimates.

Multicollinearity diagnostics revealed VIF values of 3.37 (TyG), 1.81 (FLI), 4.70 (VAI), and 6.44 (LAP). The moderate VIF for LAP reflects shared waist circumference and triglyceride components with TyG and VAI; this is expected in a composite model incorporating correlated metabolic indices and does not invalidate the cross-validated performance estimates (Figure 3F). Decision curve analysis confirmed that the composite score provided superior net benefit compared with TyG, LAP, treat-all, and treat-none strategies across the clinically relevant threshold probability range of 0.10–0.50 (Figure 3E).

### 3.5. DeLong Pairwise Comparisons

**Figure 4:**
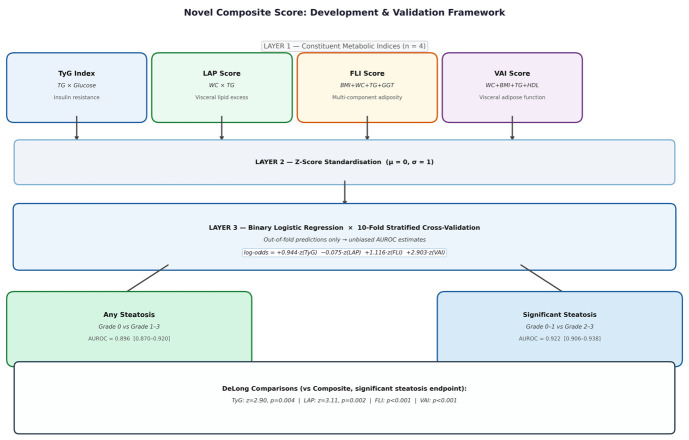
Conceptual framework for development of the novel composite score. Four metabolic indices capturing distinct pathophysiological axes (insulin resistance, visceral lipid storage, multi-component adiposity, visceral adipose).

Pairwise DeLong testing confirmed that the composite score significantly outperformed all four individual indices for the detection of significant steatosis: vs. TyG (z = 2.90, *p* = 0.004), vs. LAP (z = 3.11, *p* = 0.002), vs. FLI (z = 12.04, *p* < 0.001), and vs. VAI (z = 6.09, *p* < 0.001). For any steatosis, the composite score was similarly superior to all four individual indices. These findings are summarised in Table 3 and Figure 3D and Figure 4.

## 4. Discussion

This study presents four principal and internally consistent findings. First, TyG, LAP, and VAI each achieved excellent discrimination for ultrasonographic hepatic steatosis (AUROC 0.83–0.88), with no statistically significant difference among them for any-steatosis detection—confirming all three as first-line metabolic steatosis surrogates in this population. Second, FLI demonstrated only a moderate performance (AUROC 0.64–0.72), consistent with its derivation in a lower-BMI Western European cohort and attenuated performance at higher BMI distributions. Third, the novel composite score achieved AUROC 0.922 for significant steatosis—significantly superior to all four individual indices (DeLong *p* < 0.001 vs. FLI and VAI; *p* = 0.004 vs. TyG; *p* = 0.002 vs. LAP). Fourth, composite score calibration was excellent, supporting its interpretation as a genuine probability estimate rather than a discriminant-only metric.

Our findings also speak to the growing interest in machine-learning approaches for noninvasive steatosis detection. Whereas such models often depend on advanced reference standards such as MRI-PDFF or liver biopsy and on inputs available mainly in specialized or research settings, the present study demonstrates that even a simple logistic composite—without machine-learning complexity—significantly improves on single-index performance in a consecutively enrolled Turkish tertiary-care population, using ultrasonography as the reference, which reflects routine clinical workflow in settings where quantitative imaging is unavailable. This complementarity strengthens the case for composite metabolic scoring as a viable, broadly applicable non-invasive stratification tool across diverse clinical contexts.

The strong performance of TyG aligns with accumulating meta-analytic evidence [18]. Ling et al. [19] demonstrated a near-linear dose–response between TyG and NAFLD risk across multiple populations and reference standards, reporting pooled AUROCs in the range of 0.79–0.87. Our observed AUROC of 0.884 for significant steatosis is at the upper bound of this range, likely reflecting the high metabolic burden of our tertiary-care population (36.1% diabetes prevalence at Grade 3). Mechanistically, TyG captures combined triglyceride excess and glucose intolerance, both of which are pathogenically upstream of hepatic fat deposition via de novo lipogenesis and ectopic lipid redistribution [16]. Consistent with our findings, a recent single-centre Turkish study on non-diabetic MAFLD patients confirmed that TyG is a robust cardiometabolic risk marker in this population [20].

The comparable performance of LAP to TyG for significant steatosis (0.883 vs. 0.884, difference non-significant by DeLong) supports its clinical utility particularly when fasting insulin—required for HOMA-IR—is unavailable, supporting its utility in resource-limited settings where insulin measurement is not routinely available [9,11]. The markedly steeper absolute gradient of LAP across grades compared with TyG (5-fold vs. 1.4-fold increase from Grade 0 to Grade 3) suggests it is particularly sensitive to the higher lipid loads characteristic of severe steatosis.

The moderate performance of FLI (AUROC 0.637 for any steatosis; 0.724 for significant steatosis) is consistent with its derivation context. FLI was originally developed in a Western European community cohort (Dionysos study) characterised by a substantially lower mean BMI than the present tertiary-care Turkish population [10]. At higher BMI values, the discriminative contribution of the BMI and waist circumference components of FLI is compressed toward the upper range of the index, attenuating inter-individual separation. Population specificity of FLI has been demonstrated in multiple external validation studies, with AUROCs ranging from 0.63 in high-BMI populations to 0.84 in lean cohorts [21,22,23,24,25,26]. The right-skewed FLI distribution observed in our cohort (Table 2) is consistent with a high-BMI population; z-score standardisation prior to model fitting effectively absorbs this skewness, as all observations fell within ±3 standard deviations of the mean.

The novel composite score is the principal methodological contribution of this work. The conceptual rationale for combining indices rests on information theory: if each individual index encodes partially distinct metabolic information, a weighted combination should recover more of the latent steatosis signal than any single index alone. Empirically, the Spearman inter-correlations among TyG, LAP, VAI, and FLI ranged from near-zero (FLI–VAI: r ≈ 0.38) to moderate (TyG–LAP: r ≈ 0.62), confirming partial independence and supporting the informational complementarity hypothesis. The logistic regression framework naturally finds the optimal linear combination weights for a given discriminative objective, and 10-fold cross-validation ensures these weights are evaluated on data unseen during optimisation—a critical requirement for unbiased performance estimation.

The magnitude of improvement—AUROC 0.922 vs. best individual 0.884, Δ = 0.038—may appear modest numerically but translates to meaningful clinical gains. At a fixed specificity of 86.0%, the composite score achieves 87.1% sensitivity compared with approximately 80% for TyG alone: this corresponds to approximately 8 additional cases of Grade ≥ 2 steatosis correctly identified per 100 patients examined. At the population level of MASLD prevalence, this translates to substantial gains in early detection and opportunity for preventive intervention.

Two demographic characteristics of the cohort merit specific discussion regarding their potential influence on diagnostic performance. The male predominance (73.3% overall; 81.1% at Grade 3) reflects the known higher prevalence and severity of steatosis in men and is consistent with tertiary-care gastroenterology and endocrinology referral patterns in Turkey [27,28]. Both LAP and VAI incorporate sex-specific formulae that adjust for the distinct waist circumference and lipid thresholds in women; their performance was therefore analytically adjusted for sex, although the relatively small proportion of women (26.7%) limits subgroup-specific inference. The high diabetes prevalence at Grade 3 (36.1%) reflects the well-established association between type 2 diabetes and advanced steatosis; because TyG directly encodes fasting glucose, its performance advantage may be partially attributable to the high diabetes prevalence in the severe steatosis group rather than to visceral fat alone. Future studies stratified by diabetes status would clarify the relative performance of insulin-resistance-based vs lipid-based indices across the glycaemic spectrum.

This study has limitations. First, although consecutive patient enrolment mitigates selection bias, the retrospective, single-centre design necessitates cautious interpretation until multi-centre external validation confirms transportability to diverse populations. The cohort was predominantly male (73.3%) and was drawn from a tertiary-care setting in which diabetes prevalence increased to 36.1% at Grade 3 steatosis. These demographic characteristics may limit direct extrapolation to female-predominant populations, community-based cohorts with lower metabolic burden, or ethnically distinct groups. External validation in publicly available datasets such as NHANES, which includes more balanced sex distributions and diverse ethnic backgrounds, would help establish the generalisability of the composite score. Furthermore, the high waist circumference values observed across all steatosis grades indicate that the study sample was predominantly overweight or obese; performance of the composite score in lean MASLD—a phenotype prevalent in Asian populations and characterised by distinct metabolic features—cannot be inferred from the present data and warrants dedicated investigation. Second, ultrasonographic steatosis grading, while representing the most widely used clinical reference standard, carries inherent operator-dependency and a detection threshold (~30% hepatic fat) that may underestimate mild steatosis; quantitative validation against CAP or MRI-PDFF remains desirable. Because this study retrospectively analysed archived ultrasonographic reports generated by multiple radiologists in routine clinical practice, a prospective inter-reader reliability assessment was not feasible; the resulting uncertainty in the reference standard classification may have introduced non-differential misclassification that could attenuate the observed AUROC estimates. Third, the composite score, derived and internally validated via 10-fold cross-validation within the derivation cohort, requires prospective testing in independent samples to confirm generalizability before clinical deployment. In particular, the stringent exclusion criteria applied to achieve a diagnostically homogeneous derivation sample—removing patients with viral hepatitis, hepatotoxic medications, and significant renal insufficiency—may have inflated the observed AUROC values relative to performance in routine clinical populations where such comorbidities frequently coexist with MASLD; validation in less selected cohorts is warranted. This study focused on two clinically defined binary endpoints (any steatosis and significant steatosis), which reflect the thresholds with greatest therapeutic relevance; the discrimination of Grade 0 from Grade 1 steatosis was not the primary analytic objective, partly because ultrasonographic sensitivity is lowest precisely in this range (hepatic fat < 30%), making any reference-standard-based binary analysis at this threshold inherently unreliable. Finally, the algorithmic combination of four metabolic indices, while capturing complementary pathophysiological axes, could potentially be refined by the incorporation of additional validated scores (e.g., METS-IR, TyG-BMI) in future iterations.

## 5. Conclusions

Among four widely used metabolic scoring indices evaluated in 1204 consecutive patients, TyG, LAP, and VAI each provided excellent non-invasive discrimination for ultrasonographic hepatic steatosis (AUROC 0.83–0.88), while FLI demonstrated moderate performance consistent with derivation-population mismatch. A novel composite score derived by 10-fold cross-validated logistic regression, integrating the complementary metabolic information encoded in all four indices, significantly outperformed every constituent index for significant steatosis detection (AUROC 0.922 [95% CI [0.906–0.938]], sensitivity 87.1%, specificity 86.0%, Youden index 0.731; DeLong *p* < 0.001 vs. FLI and VAI; *p* = 0.004 vs. TyG; *p* = 0.002 vs. LAP). The composite score was well-calibrated and demonstrated robust performance across all 10 cross-validation folds. Population-level standardisation parameters (Table 4) are provided to facilitate immediate clinical application. Pending external prospective validation, this composite score may serve as a practical, entirely non-invasive, and clinically deployable tool for hepatic steatosis risk stratification in primary and secondary care settings.

## Figures and Tables

**Table 1:** Baseline clinical and biochemical characteristics stratified by ultrasonographic hepatic steatosis grade (*n* = 1204).

Characteristic	Grade 0 (*n* = 185)	Grade 1 (*n* = 539)	Grade 2 (*n* = 311)	Grade 3 (*n* = 169)	*p* Value
Age (years)	36.2 ± 11.5	41.9 ± 12.0	43.8 ± 12.4	47.1 ± 12.8	<0.001
Male sex, *n* (%)	114 (61.6)	427 (79.2)	205 (65.9)	137 (81.1)	<0.001
Diabetes mellitus, *n* (%)	2 (1.1)	46 (8.5)	52 (16.7)	61 (36.1)	<0.001
Fasting glucose (mg/dL)	89.4 ± 11.2	96.2 ± 17.7	104.8 ± 31.2	135.8 ± 71.5	<0.001
Triglycerides (mg/dL)	83.5 ± 45.4	119.0 ± 39.4	171.2 ± 55.9	273.3 ± 114.8	<0.001
HDL cholesterol (mg/dL)	53.5 ± 12.1	51.0 ± 12.0	47.6 ± 11.6	42.6 ± 15.2	<0.001
LDL cholesterol (mg/dL)	111.9 ± 27.0	128.3 ± 33.7	134.4 ± 38.4	143.3 ± 43.1	<0.001
ALT (U/L)	22.0 ± 14.4	24.0 ± 14.0	25.1 ± 15.1	30.7 ± 22.0	<0.001
AST (U/L)	19.9 ± 7.3	20.8 ± 9.5	21.3 ± 9.2	24.2 ± 16.6	0.006
GGT (U/L)	17.8 ± 9.8	22.9 ± 14.9	26.4 ± 20.6	35.4 ± 26.1	<0.001
Waist circumference (cm)	99.8 ± 11.9	106.8 ± 13.1	112.7 ± 13.6	116.6 ± 13.3	<0.001
BMI (kg/m^2^)	27.4 ± 4.2	36.4 ± 7.4	38.2 ± 7.1	41.0 ± 7.1	<0.001

Values are mean ± SD for continuous variables or n (%) for categorical variables. Continuous variables compared by Kruskal–Wallis test; categorical variables by chi-square test. ALT, alanine aminotransferase; AST, aspartate aminotransferase; GGT, gamma-glutamyltransferase; HDL, high-density lipoprotein; LDL, low-density lipoprotein.

**Table 2:** Metabolic index values stratified by ultrasonographic hepatic steatosis grade.

Score (Spearman r)	Grade 0 (*n* = 185)	Grade 1 (*n* = 539)	Grade 2 (*n* = 311)	Grade 3 (*n* = 169)	*p* Value
TyG index (r = 0.729)	8.01 (7.84–8.25)	8.60 (8.38–8.79)	9.05 (8.84–9.25)	9.55 (9.28–9.97)	<0.001
LAP score (r = 0.715)	29.72 (18.63–45.12)	53.74 (39.08–71.25)	91.96 (71.07–116.77)	150.66 (119.50–190.04)	<0.001
FLI score (r = 0.371)	2.15 (0.79–71.00)	4.60 (1.96–11.56)	12.79 (5.70–87.28)	19.27 (12.83–35.70)	<0.001
VAI score (r = 0.660)	1.55 (1.23–2.10)	3.18 (2.37–4.11)	5.32 (3.19–6.72)	9.95 (7.07–12.50)	<0.001

**Table 3:** Diagnostic performance of metabolic indices and the novel composite score—ROC analysis results.

Score	AUROC—Any Steatosis (95% CI)	AUROC—Significant Steatosis (95% CI)	Sen. (%)	Spec. (%)	Youden Index	DeLong *p* vs. Composite
TyG index	0.870 [0.837–0.900]	0.884 [0.863–0.904]	80.8	84.9	0.658	<0.001
LAP score	0.846 [0.812–0.879]	0.883 [0.862–0.901]	77.3	84.0	0.613	<0.001
FLI score	0.637 [0.582–0.689]	0.724 [0.694–0.752]	82.9	58.4	0.413	<0.001
VAI score	0.878 [0.854–0.899]	0.827 [0.800–0.854]	68.3	91.4	0.598	<0.001
**Novel Composite Score**	**0.896 [0.870–0.920]**	**0.922 [0.906–0.938]**	**87.1**	**86.0**	**0.731**	**Reference**

AUROCs for the composite score are based exclusively on 10-fold cross-validated, out-of-sample predictions; 95% CIs from 1000 bootstrap iterations. Sensitivity and specificity are reported at the Youden-optimal cut-off for detection of significant steatosis (Grade ≥ 2). DeLong *p* values represent pairwise comparisons of each individual index against the novel composite score for significant steatosis. The composite score row is highlighted. Bold values denote the novel composite score, which achieved the best diagnostic performance. FLI, Fatty Liver Index; LAP, Lipid Accumulation Product; Sen., sensitivity; Spec., specificity; TyG, Triglyceride–Glucose index; VAI, Visceral Adiposity Index. Functions are standardised and combined via logistic regression with 10-fold cross-validation, yielding the composite score evaluated against two binary endpoints.

**Table 4:** Population parameters for z-score standardisation of the composite score predictors (derivation cohort, *n* = 1204).

Index	Mean (μ)	SD (σ)	z-Score Formula
TyG index	8.78	0.62	(TyG − 8.78)/0.62
LAP score	78.24	52.1	(LAP − 78.24)/52.1
FLI score	28.71	36.39	(FLI − 28.71)/36.39
VAI score	4.5	3.83	(VAI − 4.5)/3.83

Composite score: log-odds = +0.9444 × z(TyG) − 0.0749 × z(LAP) + 1.1156 × z(FLI) + 2.9025 × z(VAI); Probability = 1/(1 + exp(−log-odds)). SD, standard deviation.

## Data Availability

The datasets analysed during the current study are available from the corresponding authors on reasonable request, subject to patient confidentiality requirements.

## Abbreviations

ALT	Alanine aminotransferase
AST	Aspartate aminotransferase
AUROC	Area under the receiver operating characteristic curve
BMI	Body mass index
CAP	Controlled attenuation parameter
CI	Confidence interval
FLI	Fatty Liver Index
GGT	Gamma-glutamyltransferase
HDL	High-density lipoprotein
IQR	Interquartile range
LAP	Lipid Accumulation Product
MRI-PDFF	MRI-derived proton density fat fraction
NAFLD	Non-alcoholic fatty liver disease
MASLD	Metabolic dysfunction-associated steatotic liver disease
NASH	Non-alcoholic steatohepatitis
ROC	Receiver operating characteristic
SD	Standard deviation
TyG	Triglyceride–Glucose index
USG	Ultrasonography
VAI	Visceral Adiposity Index
